# Prognostic utility of lncRNAs (LINC00565 and LINC00641) as molecular markers in glioblastoma multiforme (GBM)

**DOI:** 10.1007/s11060-022-04030-7

**Published:** 2022-06-06

**Authors:** Rehab G. Amer, Lobna R. Ezz El Arab, Dalia Abd El Ghany, Amr S. Saad, Nermean Bahie-Eldin, Menha Swellam

**Affiliations:** 1 Clinical Oncology Department, Ain Shams University, Cairo, Egypt; 2 Biochemistry Department, Biotechnology Research Institute, High Throughput Molecular and Genetic laboratory, Central Laboratories Network and the Centers of Excellence, National Research Centre, Dokki, Giza, Egypt

**Keywords:** lncRNA, Glioblastoma multiforme, Prediction, Prognosis, Response

## Abstract

**Background and aim:**

Glioblastoma multiforme (GBM) is primary brain tumor grade IV characterized by fast cell proliferation, high mortality and morbidity and most lethal gliomas. Molecular approaches underlying its pathogenesis and progression with diagnostic and prognostic value have been an area of interest. Long-non coding RNAs (lncRNAs) aberrantly expressed in GBM have been recently studied. The aim is to investigate the clinical role of lncRNA565 and lncRNA641 in GBM patients.

**Patients and methods:**

Blood samples were withdrawn from 35 newly diagnosed GBM cases with 15 healthy individuals, then lncRNA565 and lncRNA641 expression were evaluated using real time-PCR. Their diagnostic efficacy was detected using receiver operating characteristic curve. Progression free survival (PFS) and overall survival (OS) were studied using Kaplan–Meier curves.

**Results:**

lncRNAs expressions were increased significantly among GBM as compared to control group. Their expressions were correlated with clinico-pathological data and survival pattern for the studied GBM patients. Higher levels of both lncRNAs were correlated to worse performance status. Expression of lncRNA565 was increased with large tumor size (≥ 5 cm). Survival analysis showed that both investigated lncRNA were increased with worse PFS and OS.

**Conclusion:**

Expression of lncRNA565 and lncRNA641 in a liquid biopsy sample can be used as prognostic biomarker for GBM patients.

## Introduction

Glioblastoma multiforme (GBM) is the most common, aggressive and poorly prognosed brain tumor that represents about 82% of primary CNS malignancy in adults. It was classified as grade IV tumor by the World Health Organization (WHO) [1] Previously, it has been reported as an age-related neurological disease [2], among Egyptian adult GBM patients has reported to be of 50 ± 2 years [3–5]. No definitive causing etiology has been found, but factors that may be related to GBM risk are: decreased susceptibility to allergy, immune factors and immune genes, and some nucleotide (nt) polymorphisms [6]. Despite of the progress has been made in the standard treatment as resection techniques, radiation therapy, and chemotherapeutic strategies, the median survival are about 15 months [7]. The WHO classification in 2016 included molecular parameters in the diagnostic schema, as it’s more relevant to outcome than histological grading alone [8]. Recently, the fifth edition of the WHO Classification of Tumors of the Central Nervous System (WHO CNS-5)—published in 2021—magnifies the molecular diagnostics role in CNS tumor classification [9]. Survival in GBM patients showed to be affected by individual molecular biomarkers [1]. So, it is important to study the molecular variability of GBMs and its effect on the biological behavior of individual GBMs. This can help to adjust methods for proper diagnosis, prognosis and therapeutic response prediction [10].

Since the biggest obstacles of cancer therapy are still present, such as treatment resistance and early recurrence; the search for novel biomarkers has steadily continued. Recently, certain molecular groups have gained increased attention. Non-coding RNAs (nc-RNAs) is one of these groups, although previously thought to be a transcriptional noise with no apparent function. They have two subtypes according to the length: small nc-RNAs are shorter than 200 nts, while long nc-RNAs (lncRNAs) are at least 200 nts long [11]. Long noncoding RNA are RNAs that lack functional protein coding ability, but they are important as players in the complex genome regulatory network, these transcripts may have prognostic or even therapeutic applications [12].

In carcinogenesis, deregulated lncRNAs can affect both oncogenic and tumor-suppressing pathways [13]. lncRNAs are present in body fluids, including serum, plasma, and urine, that allow tracking of the cancer genome without need of a tissue biopsy especially non accessible sites. A variety of studies demonstrate the role of circulating lncRNAs as biomarkers for cancer detection and prognosis in multiple cancers [14].

Some of dysregulated lncRNAs have been reported to play oncogenic and tumor suppressive roles in cancer development, progression, and metastasis. For example: *HOTAIR*, *PVT1*, *H19* and *MALAT1* showed oncogenic effect, while *MEG3* and *GAS5* have tumor suppressor effect [15]. Through bioinformatics analysis, it was identified that lncR 641 is significantly down-regulated in bladder cancer tissues, while lncR 641 overexpression suppresses proliferation, migration and invasion of bladder cancer [16]. In addition, it was demonstrated that lncR 641 can function as a tumor suppressor in non-small-cell lung cancer (NSCLC) via a competing endogenous RNAs (ceRNA) network [17]. Whereas it was reported that, lncR 565 promoted the progression of ovarian cancer via upregulating GAS6, which has been confirmed to promote tumor progression [18].

In GBM, controlling lncRNAs are under investigated, several studies aimed to identify lncRNAs that could serve as diagnostic, prognostic or predictive markers and correlated them with tumor progression and survival [10]. As example, CASC2, TSLC1-AS1, and ADAMTS9-AS2 are tumor suppressor lncRNAs, while linc-POU3F3, HOTAIR, and H19 function to promote GBM cell cycle progression [19].

Zhang and his colleagues reported a set of lncRNAs (C20orf166-AS1, lncR 645, LBX2-AS1, lncR 565, lncR 641, and PRRT3-AS1) that have prognostic value for GBM [17]. By lncRNAs bioinformatics analysis using Cancer Genome Atlas (TCGA) and formed a risk scoring model based on these lncRNAs to divide the patients into high and low-risk groups. It showed that the low-risk group had significantly longer OS compared to the high-risk group (16.61 ± 14.22 months vs 9.83 ± 6.17 months, *P* = 0.000127) [20].

Moreover, another study showed significant prognostic value of six-lncRNA signature (LBX2-AS1, lncR 641, PRRT3-AS1, and lncR 565) and significant association with focal adhesion, extracellular matrix (ECM) receptor interaction, and mitogen-activated protein kinase (MAPK) signaling pathways. These four pathways showed involvement of twelve common genes, including TGFB 1 gene [19].

Using liquid biopsy samples for detection of tumor cells has been widely studied [21, 22], because they are clinically useful as minimally invasive approach for early detection of cancer and may be helpful as follow-up markers since they can be present in detectable concentrations [15]. lncRNAs are among the circulating biomolecules that can be studied in liquid samples. Recent studies were concerned about detection of lncRNAs in blood samples from solid tumors [23, 24]. The current study aims to assess the clinical role of two long noncoding RNAs: long intergenic non-protein coding RNA 641 (lncRNA-641) and long intergenic non-protein coding RNA 565 (lncRNA-565) as minimal invasive molecular markers for GBM and detect their role as prognostic liquid biomarkers.

## Patients and methods

The study was conducted on patients presented to Clinical Oncology Department, Faculty of Medicine, Ain Shams University from September 2019 to October 2021. After obtaining the approval from the Ethical Committee and individuals or their first degree relatives or legal guardians signed the informed consents.

### Enrolled individuals

Blood samples were collected from newly diagnosed adult glioblastoma (GBM) patients (n = 35) whom fulfill the following inclusion criteria: newly diagnosed GBM cancer patients with no history of other malignancy, age more than 18 years, with performance less than or equal 2, according to the ECOG (the Eastern Cooperative Oncology Group) [25] and samples were withdrawn before they have received any oncological treatment strategies. Any GBM patients whom did not fulfill the inclusion criteria were excluded from the study. As a control group, 15 healthy individuals were enrolled in the study; both groups were of matched ages.

### Sample collection and processing

Three ml of blood samples were collected from all individuals in gel vacutainer tubes (Greiner Bio-One GmbH, Kremsmünster, Austria) and transferred to lab within 30 min for lncRNAs detection. After centrifugation of blood samples at 10,000 × g for 10 min at 4 °C (3-18KS, Sigma, Germany) the sera were separated and stored into aliquots and stored immediately in RNase free tubes at − 80 °C till further processing.

### lncRNA extraction

lncRNA extraction was performed using miRNeasy Mini kit (Catalogue # 217004, Qiagen, USA). Briefly, 5 vol QIAzol lysis reagent (RNA extraction reagent) were added to a volume of the thawed serum sample, and then vortexed was applied. Lysates were left for 5 min at room temperature (25 °C) to promote dissociation of nucleo-protein complexes. Phase separation step was executed by adding chloroform in a ratio of 1:1 volume of starting sample to the starting sample to the tube containing the lysate, and vortex was applied followed by centrifugation at 12,000 × g for 15 min (4 °C). The upper aqueous phase was transferred to a new collection tube. Afterwards, 1.5 vol of 100% ethanol were added to the aqueous phase followed by pipetting up and down several times. The sample (up to 700 μl) was transferred into an RNeasy Mini spin column in a 2 ml collection tube and then centrifuged for 15 s at ≥ 8000 × g for 15 min at room temperature. The RWT buffer (700 μl) was added to the RNeasy Mini spin column. After centrifugation, the flow-through was discarded and RPE buffer (500 μl) was added and the column was centrifuged and flow-through was discarded. The RPE step was repeated. The RNeasy Mini spin column was placed into a new 2 ml collection tube and centrifuged at full speed (14,000 × g) for 2 min. Finally, the RNeasy Mini spin column was transferred to a new 1.5 ml collection tube. RNase-free water (30 μl) was directly added onto the RNeasy Mini spin column membrane and centrifugation was done for 1 min at ≥ 8000 × g. The purity and the concentration of the purified lncRNA was detected using spectrophotometer Nano-drop (Quawell, Q-500, Scribner, USA) and stored at − 80 °C till further assessments.

### Reverse transcription and cDNA preparation

Reverse transcription of lncRNA was carried out using RT^2^ First Strand Kit (Cat number # 330404, Qiagen, USA) as recommended in the manufacturer's instructions by using a total volume of 10 μl of reverse transcription reaction components. The polymerase chain reaction (PCR) tubes were then placed in thermal cycler (SureCycler 8800, Agilent, USA) and the transcription profile was adjusted for 60 min at 37 °C. Complementary DNA purity and concentration was detected using spectrophotometer Nano-drop (Quawell, Q-500, Scribner, USA) and stored at − 20 °C till performing qPCR.

### Quantitative real-time PCR (qPCR)

Quantitative real-time PCR was performed using miScript primer assay (Cat number 330701, Qiagen, USA) for lncR 565 (Accession no. LPH23084A), and for lncR 641 (Accession no. LPH21254A) the reaction was carried out using RT^2^ qPCR SYBR Green (Cat number 330520, Qiagen, USA). Their expression was normalized using *β-Actin* (Accession no. LPH28471A) as a housekeeping gene. The reactions for lncRNA primer assays were carried out by using cDNA with concentrations adjusted to 2 ng/PCR reaction, and a total volume of 25 μl, whereas cycling conditions were: heating step at 95 °C for 10 min where HotStart DNA *Taq-*polymerase was activated, followed by 40 cycles for denaturation at 95 °C for 15 s, then annealing at 55 °C for 40 s. Extension was then performed at 72 °C for 30 s, in which fluorescence was acquired and detected by Stratagene Real-time PCR system (Max3005P QPCR system, Stratagene, Agilent biotechnology, USA). The relative expression levels of the investigated lncRNAs were evaluated using the 2^−ΔΔ*Ct*^ method. The cycle threshold (*Ct*) value is the number of qPCR cycles required for the fluorescent signal to cross a specified value. The relative expression levels of the investigated lncRNAs were evaluated using the 2^−ΔΔ*Ct*^ method [26]. Δ*Ct* was calculated by subtracting the *Ct* values of *β-Actin* from *Ct* of investigated lncRNA for detection of its expression, then ΔΔ*Ct* was calculated by subtracting the Δ*Ct* of the control samples from the Δ*Ct* of the GBM samples, after words 2^−ΔΔ*Ct*^ (fold change expression) was calculated for lncRNAs under investigation.

### Treatment strategy

All GBM patients were assessed clinically (by complete history, clinical examination and neurologic examination) and with brain imaging in order to receive their standardized treatment protocol, which involve maximum safe resection (if possible), followed by radiotherapy conventional fractions (total dose of 60 Gy, given 2 Gy per fraction for 30 fractions over 6 weeks) or hypofractionation (45 Gy in 15 fractions over 3 weeks) with concomitant temozolomide (TMZ) chemotherapy (75 mg/m^2^ every day till end of radiotherapy) with regular follow up, then re-evaluated clinically and radiologically followed by adjuvant six cycles of TMZ treatment at a dose of 150 mg/m^2^ body surface area from days 1 to 5 every 28 days with clinical monitoring.

During regular clinical follow up, patient were assessed by gadolinium-enhanced magnetic resonance imaging (Gd-MRI) 45 days after RT and then performed every 3 months or at time of clinical evidences of neurologic progression. Tumor response was evaluated on the basis of radiological RANO response criteria (2010) [27]. Complete response (CR): disappearance of all known brain lesion. Partial response (PR): 50% or greater decrease in measurable brain lesion or an objective improvement in evaluable brain lesion. Stable disease (SD): brain lesion unchanged (< 50% decrease or < 25% increase in the size of measurable lesions). Progressive disease (PD): ≥ 25% increase in size of some or all of brain lesions and/or the appearance of any new brain lesions. Another risk factor was steroid dependency: it is defined as failure to taper steroids after radiotherapy course or increasing the dose. It indicates bad prognosis in GBM patients.

### Statistical analysis

Data was analyzed using SPSS (version 12 SPSS, Inc., Chicago USA) and P-value were two-tailed and considered significant if < 0.05. The fold change in investigated lncRNAs was calculated using the equation of 2^−*ΔΔCt*^. The association between the clinico-pathological and demographic factors with investigated lncRNAs was determined by ANOVA analysis. Receiver operating characteristic (ROC) curve was plotted between GBM patients and healthy individuals to detect the sensitivities and the specificities for the lncRNAs and their clinical efficacy [28]. Progression free survival (PFS): it’s time from start of enrollment till progression of the disease proved by MRI imaging or clinical deterioration. Overall survival (OS): was calculated from the date of random assignment to the date of death or lost follow up, both were analyzed using Kaplan–Meier statistical method and compared by log-rank test.

## Results

**Fig. 1:**
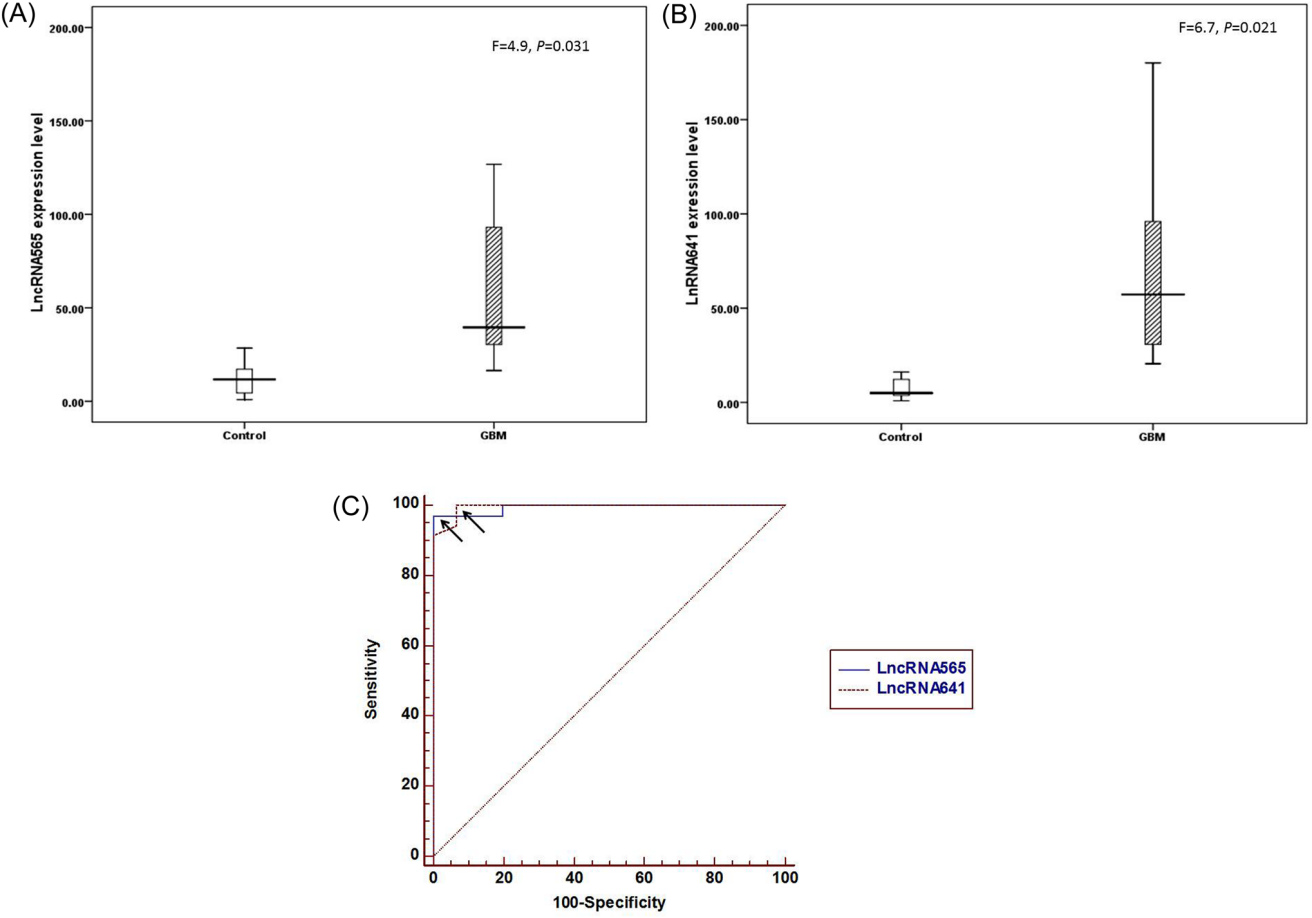
**A** Box plot comparing lncRNA565 expression level in control group versus GBM, **B** box plot comparing lncRNA641 expression level in control group versus GBM, and **C** receiver operating characteristic curve (ROC curve) for investigated lncRNAs. Arrows donate to the optimal cutoff point that discriminates between GBM cases and control individuals

The studied cohorts were divided into two groups: newly diagnosed GBM patients (n = 35) with an age range (22–62), 26 of them (74.3%) were below 60 years with a mean age of (47.7 ± 1.2 years), 24 of them (68.6%) were males; the second group was healthy volunteers serve as control group (n = 15) with an age range (22–58), with a mean age (33.9 ± 8.1 years), 9 of them (60%) were males. According to the performance status, 16 patients had (ECOG < 2), while other patients had (ECOG = 2). Medical comorbidities reported as 6 diabetic, 4 hypertensive, 3 viral hepatitis (HCV) patients and 5 with other medical comorbidities (asthma, hypothyroidism). Positive family history of GBM was reported in 3 patients. The main presenting symptoms were increased ICT, neurological deficit and convulsions (N = 23, 12, 13, respectively). As regard to the tumor criteria, 22 patients had left sided lesion, 17 had right sided lesion, as multiple lesions were reported in 5 patients. According to tumor lobe, 7 cases had frontal lobe lesions, 26 cases had non-frontal lesions (tempo-partial and posterior-fossa) and 2 cases had lesions in both frontal and non-frontal areas. Tumor size was initially ≥ 5 cm in 22 patients. Diagnosis of GBM was pathologically confirmed in 27 cases [after biopsy (N = 17) or surgical resection (N = 10)], while MRS was used in diagnosis of 8 cases with non-accessible sites. Clinico-pathological characteristics for GBM cases were summarized in Table 1. The estimated mean expression of lncR 565 for GBM patients group [mean ± standard error of mean, SEM = 106 ± 28.3) and (9.2 ± 1.6) for control group. While lncR 641 mean expression was (89 ± 20) for GBM group and (8.1 ± 1.8) for control group. That showed statistically significant differences between GBM group and control group (at *P* value = 0.031, and 0.021) for lncR 565 and lncR 641 levels, respectively, as shown in Fig. 1A, B. Their diagnostic efficacy as were determined by plotting ROC curve (Fig. 1C). Accordingly, significant difference was reported between GBM and control. Moreover, the diagnostic efficacy (sensitivity and specificity) revealed 97% and 100% for lncR 565 at AUC equals 0.994 with a cutoff point 19.18 and 100%, 93.3% for lncR 641 with AUC equals 0.995 with a cutoff point 16.16.

**Table 1:** Clinico-pathological characteristics of GBM patients (N = 35)

Character	N, (%)
Comorbidities	
DM	6, (17.1%)
HTN	4, (11.4%)
Viral hepatitis	3, (8.6%)
Others	5, (14.3%)
Irrelevant	18, (51.4%)
Family history	
GBM	3, (8.6%)
Other tumors	5, (14.3%)
ECGO	
< 2	16, (45.7%)
= 2	19, (54.3%)
Presenting symptoms	
ICT	23, (65.7%)
Neurological deficient	12, (34.3%)
Convulsions	13, (37.1%)
Tumor site	
Rt site	17, (48.6%)
Lt site	22, (62.9%)
Multiplicity	5, (14.3%) (4 Bilateral and 1 unilateral)
Site of primary lesion	
Frontal	7, (20%)
Non-frontal (temporo-parietal and posterior fossa)	26, (74.3%)
Both (frontal and non-frontal)	2, (5.7%)
Tumor size	
< 5 cm	13, (37.1%)
≥ 5 cm	22, (62.9%)
Diagnosis	
MRS	8, (22.9%)
Pathology (intervention)	27, (77.1%)
Type of intervention	(N = 27)
Biopsy	17, (63%)
Resection	10, (37%)
a Gross total resection	4, (40%)
b Subtotal resection	6, (60%)

*DM* diabetes mellitus, *HTN* hypertension, *MF* medically free, *FM* family history, *ECGO* performance status, *ICT* increased intracranial tension, *Rt* right, *Lt* left, *MRS* magnetic resonance spectroscopy

Using non-parametric analysis by Mann–Whitney and Kruskal–Wallis Test the expression levels of investigated lncRNAs with clinical criteria for enrolled GBM cases were detected. The impact of lncR 565 and lncR 641 on demographic characteristics reported no significant difference between age, gender, family history but statistically significant higher mean levels were reported with performance status equals 2 (*P* = 0.04 and 0.03, respectively), presentation with fits was associated with significantly lower mean level (*P* = 0.001 and 0.02, respectively). A part from lncR 641, significantly higher mean lncR 565 level was reported in patients presented with increased intracranial tension (*P* = 0.002) and tumor size > 5 cm (*P* = 0.04).

Upon treatment, as summarized Table 2, 32 cases received radiotherapy (CCRTH). Conventional fractions were used for treatment of 18 cases, while the remaining received hypo-fractionated radiotherapy. Conformal (3D) technique of radiotherapy was used in 23 cases, while IMRT was used in 9 cases. Subsequently, 21 patients received adjuvant chemotherapy. As regards toxicity, it was tolerable and manageable; radiotherapy toxicity (neurocognitive symptoms, dermatitis, alopecia, otitis media) was reported as acute toxicity (≥ G3) in 12 cases and late in 3 cases. While for chemotherapy toxicity, 11 patients reported G1 and G2 (most commonly of GIT symptoms) and G3 toxicity (heamatological) only in 1 patient. The estimated lncR 565 and -641 expression mean levels in radiotherapy treated patients were (mean rank = 110.7, and 88.84, respectively). Significantly higher levels of lncR 641 were associated with steroid dependence (N = 22) and (*P* = 0.011).

**Table 2:** Expression level of lncRNA565 and lncRNA641 in GBM patients in relation to received treatment

Received treatment		N (%)	2^−∆∆Ct^ lnRNA-565	2^−∆∆Ct^ lnRNA-641
			Mean	Mean
CCRTH (TEMODAL)	No	3 (8.57%)	18.6	18.3
	Yes	32 (91.42%)	17.7	17.89
RTH fractions	Conventional	18 (56.25%)	16.8	18.4
	Hypofractionated	14 (43.75%)	17.9	17.8
Rth technique	3D conformal	23 (71.87%)	12	12
	IMRT	9 (28.12%)	24.5	24
			P = 0.003	P = 0.007
Adjuvant CTH	No	11 (34.37%	18.6	17.8
	Yes	21 (65.62%)	16.5	15.4
Steroid dependence	No	10 (31.25%)	12.3	10.3
	Yes	22 (68.75%)	18.41	19.32
				P = 0.011

*CCRTH* concurrent chemo-radiotherapy, *CTH* chemotherapy, *RTH* radiotherapy

According to treatment response, patients were categorized into CR (2), PR (5), SD (11) and disease progression (PD = 14), but they did not show significant correlation with investigated lncRNAs.

Survival analyses showed that after median follow up of (10 months) for 32 GBM patients, the estimated median PFS was 11 months and median OS was 13 months, 3 lost follow up (1 COVID related death during radiotherapy planning and 2 others were lost follow up on first week of radiotherapy), they were excluded from treatment and survival analysis.

**Fig. 2:**
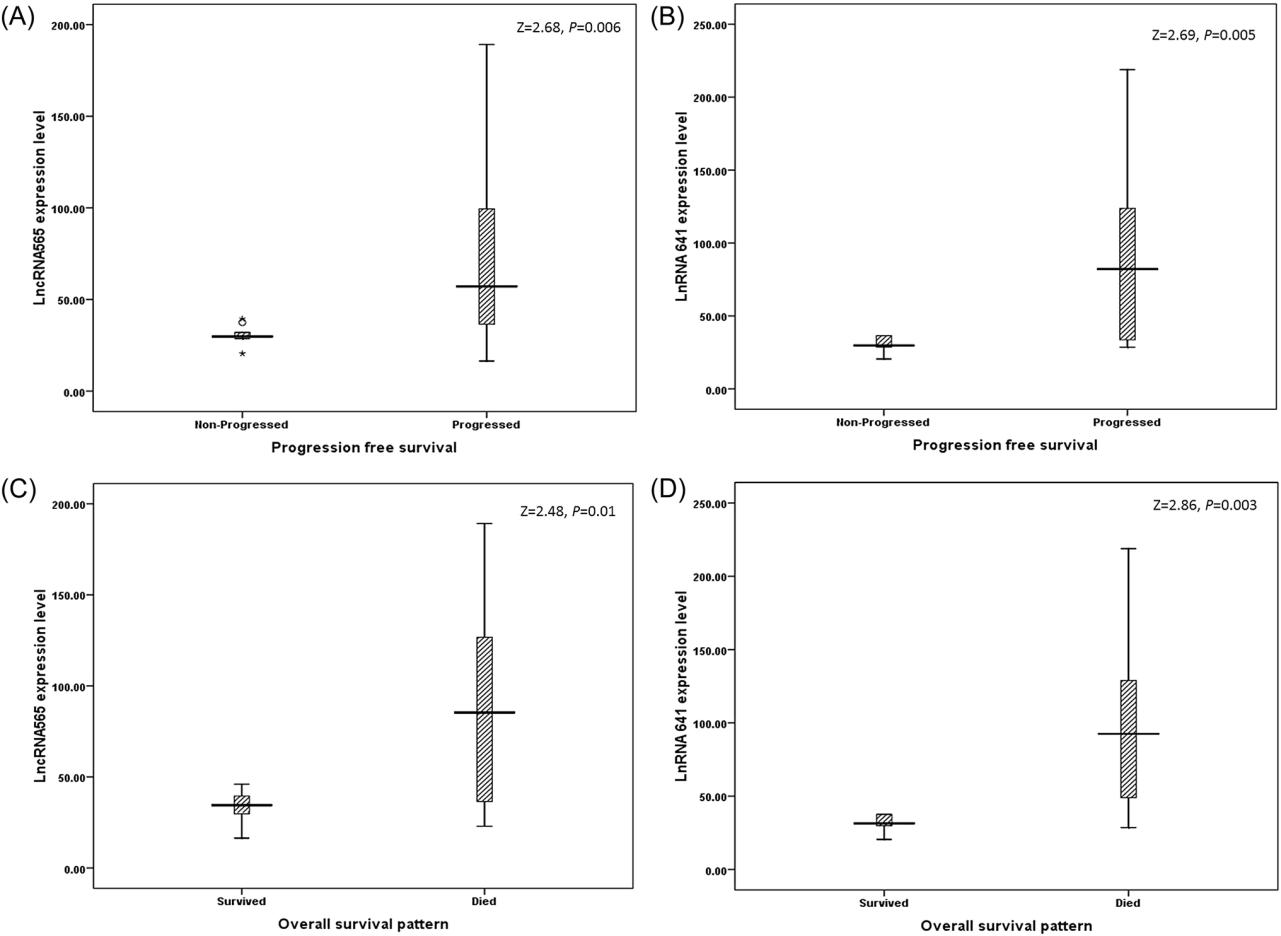
Distribution of lncRNAs versus follow-up pattern (median follow up 10 months). Progression free survival among GBM cases as 9 cases reported free survival and 26 GBM were progressed for (**A**) lncRNA565 and (**B**) lncRNA641. Overall survival pattern among GBM cases as 14 GBM cases survived and 18 GBM were died for **C** lncRNA565 and **D** lncRNA641

After follow up, mean rank levels for lncRNAs expression reported significant higher levels in relation to progressed cases as shown in Fig. 2A, B and death as shown in Fig. 2C, D.

The mean levels of lncRNAs in GBM patients were 97 for lncR 565, and 89 for lncR 641. Accordingly, the expression level for lncRNAs were grouped into high (above the mean value) and low (blow or equal the mean value). Kaplan–Meier curves were used to detect the relation between investigated lncRNAs and survival pattern.

**Fig. 3:**
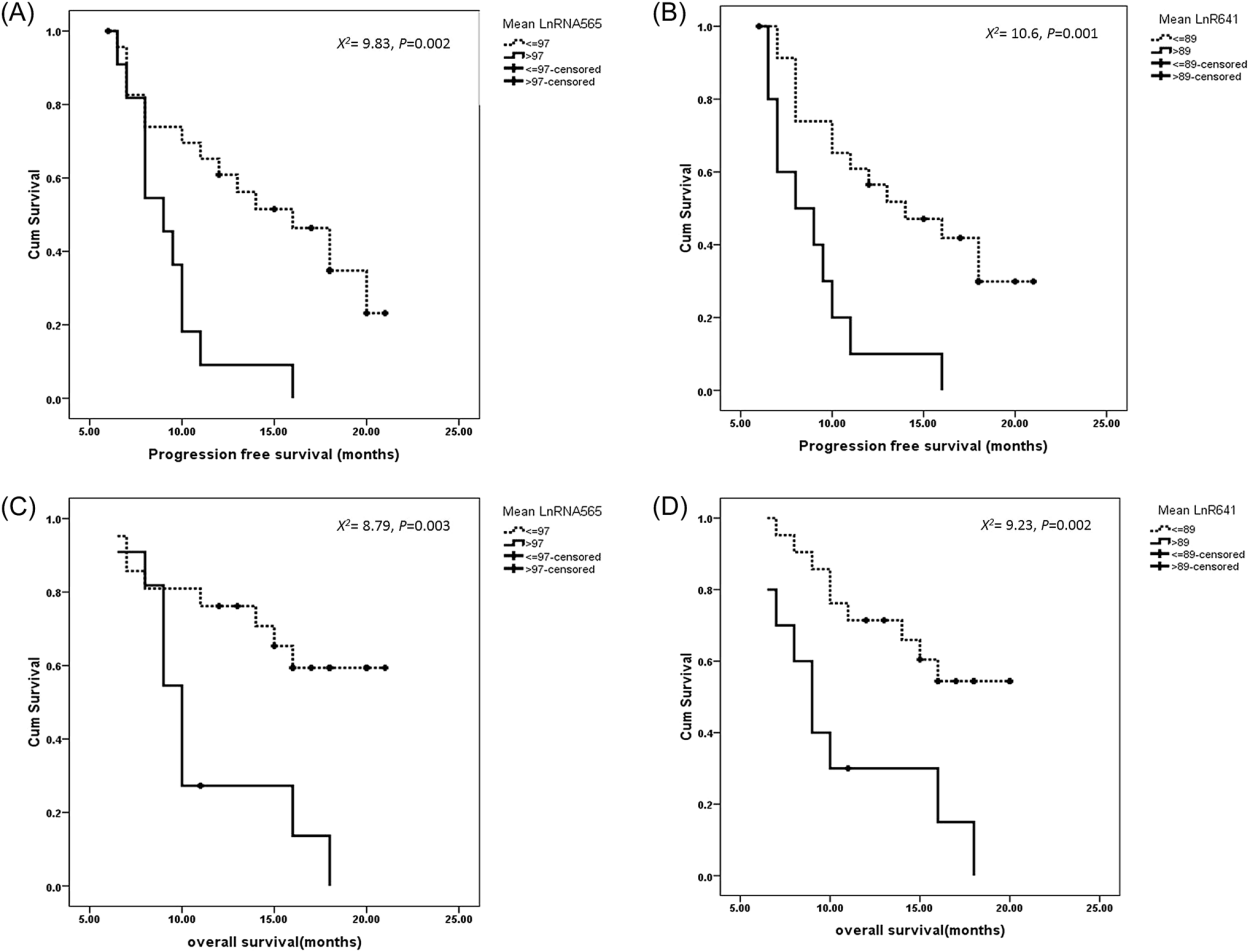
Survival patterns for investigated lncRNAs, as **A** progression free survival for lncRNA565 and **B** PFS for lncRNA641 and both reported to be significant, **C** overall survival for lncRNA565 and **D** OS for lncRNA641 and both reported to be significant

Significant difference was reported between lncR 565 and lncR 641 with PFS. GBM patients with high expression levels reported worse PFS (*P* = 0.002 and 0.001, respectively) as shown in Fig. 3A, B when compared to their counterparts with low expression levels. Similarly, expression of lncR 565 and lncR 641 reported significant differences with OS, where GBM patients with worse OS showed significantly higher expression levels (*P* = 0.003 and 0.002, respectively), as presented in Fig. 3C, D. Notably, patients underwent gross total resection (N = 4) showed longer PFS (24, 24, 22, 20 months, respectively) and OS (25, 24, 24, 20 months, respectively).

Both lncRNA were significantly correlated with each other when all individuals (n = 50) were considered (*X*^2^ = 0.825, *P* < 0.0001) and when GBM cases (n = 35) where considered (*X*^2^ = 0.805, *P* < 0.0001).

## Discussion

Growing evidence designates the association between dysregulation of lncRNAs expression with various types of cancer [19, 29], including GBM [30]. Moreover their accessibility to be detected in liquid samples made them of great value for diagnosis, prediction of prognosis and follow-up molecular [31]. In previous studies it has been reported that biological and physiological roles of lncRNAs have been investigated under limelight that their expression takes part in many biological processes and their aberrant expression is confirmed in gliomas. The recent researches on lncRNAs have reported that intergenic nc-RNA can act as endogenous miRNA sponge in cancer cells to promote tumor development [32]. Hence molecular classification based on expression of lncRNAs may direct for gene therapy and may aid to a better procedure for targeted therapy selection [33, 34].

In the current prospective study, two lncRNAs were investigated (lncR 565 and lncR 641) in blood samples from GBM patients and a healthy control group were recruited. lncR 565 reported significant increase in GBM patients as compared to control group; moreover it has revealed a good diagnostic efficacy with 97% sensitivity and absolute specificity and the AUC was reported to be 0.994 at cutoff point (19.18-fold expression). This result indicates that lncR 565 is aberrantly up-regulated in GBM cases and can be utilized as liquid molecular marker for diagnosis of GBM. Similarly, lncR 641 reported significant increase in GBM cases as compared to control ones with diagnostic efficacy of absolute sensitivity and 93.3% specificity. These findings indicate the usefulness of lncRNA641 as liquid molecular marker that can aid in diagnosis of GBM.

Alterations of lncR 565 and lncR 641 in GBM have been rarely investigated. Both were part of a recent risk scoring model using lncRNAs in GBM patients was constructed to divide them into different risk groups with significantly different survival rates. In addition, the study pointed that both lncRNAs showed predictive value for patient’s survival. Aberrant altered levels of lncR 565 and lncR 641 have been linked to ECM receptor interaction, focal adhesion and MAPK signaling pathways [20], that is involved in controlling GBM cell proliferation and migration [35].

Regarding to the current study, the relation between expression levels of both lncRNAs (lncR 565 and lncR 641) and the clinical data of studied cases, their levels reported significant change with worse performance status (= 2), also lncR 565 mean level was significantly increased with patients reporting tumor mass ≥ 5 cm indicating that both lncRNAs may be linked to tumor aggressiveness. Although it has been reported earlier that GBM is an age-related neurological disorder in adults [2], current data did not report significant difference between investigated lncRNAs and age of GBM cases.

Given the sizable studies of the role for lncRNAs cellular processes [34, 36] made them important to be considered in the context of GBM heterogeneity and treatment resistance. In the current study the median follow-up for GBM patients was 10 months and the estimated median PFS was 11 months and median OS was 13 months which concurrent with the median follow up period of previous studies on GBM [37, 38]. Survival pattern analysis showed higher levels of lncR 565 and lncR 641 reported significant relation to worse PFS and OS. These findings revealed the impact role of lncRNAs as minimally non-invasive liquid biopsy marker to be used as predictor for prognosis. The current finding is consistent with reported data from Liang and his colleagues who confirmed that lncR 565 and lncR 641 reported prognostic signature for GBM cases using several bioinformatics approaches [19].

In conclusion, to our knowledge this is the first study to detect lncR 565 and lncR 641 in liquid biopsy samples from Egyptian GBM patients although with some limitation due to small number of sample but it gives a shed on the importance of the clinical impact of these investigated lncRNAs as both diagnostic and prognostic markers which may aid in the future for RNA-based therapeutics.

## Data Availability

Author elects not share data.
